# A Systematic Review of Models Used and Preferences for Continuing Education and Continuing Professional Development of Pharmacists

**DOI:** 10.3390/pharmacy7040154

**Published:** 2019-11-16

**Authors:** Ricarda Micallef, Reem Kayyali

**Affiliations:** Pharmacy Department, Kingston University, Kingston Upon Thames KT1 2EE, UK; r.micallef@kingston.ac.uk

**Keywords:** pharmacist, models, continuing education (CE), continuing professional development (CPD), systematic review

## Abstract

Continuing Education (CE) or Continuing Professional Development (CPD) are used by pharmacists globally to maintain up-to-date knowledge and skills throughout their careers. The primary aim of this study was to identify the formats or models used by pharmacists for CE and CPD globally. The secondary aim was to identify preferences of pharmacists, in relation to the variety of formats or models used to fulfil mandatory requirements, in order to support future planning of lifelong learning events. A systematic review was performed using PubMed, Science Direct, and Web of Science covering a time period from 1995 until March 2018. Searches were conducted in English, with studies on undergraduate studies being excluded. Eighteen papers from an initial search of 4561 were included from 2004 to 2014. All studies focused on pharmacists. Three studies identified face-to-face learning as a preference, with six studies identifying a positive impact of interactive learning. All four identified studies focusing on online provision were linked to CE. One study highlighted the benefits of blended learning. Two studies identified concluded that no one size fits all. A clear structure of event was highlighted in three studies. Three studies highlighted the relevance of topics to practice, and two studies showed the need for opportunities to apply knowledge. Due to the variety of formats and no consistent model, no perfect model or activity has been identified. However, CPD showed increased practice outcomes versus CE. Although an increasing amount of technology is being utilized, face-to-face learning is still preferred. Interactive, multiple-format learning should be used where possible, to reflect preferences of different learners. There is a need for a structured approach to the planning and learning event itself to support CE and CPD.

## 1. Introduction

Continuing Professional Development (CPD) has been mentioned in pharmacy since the early 2000s, both in the United States and Great Britain [1,2]. CPD is required to ensure practitioners are up-to-date with current drugs and guidelines, and to ensure they are providing optimal patient care. CPD is self-directed, and supports the maintenance of knowledge, skills, and behaviors required for effective personal practice [2]. With increasing new roles for pharmacists and other healthcare professionals, pharmacists need to be trained to ensure service provision and competence, wherever they work [3]. This knowledge needs to be updated regularly to keep up to date with the changing role, with better critical thinking and collaboration [4]. When completing CPD, it is important for the healthcare professional to recognize not just the “how”, but also the “why” [5,6].

Continuing Education (CE) has been around for longer than CPD globally and is still the mainstay of post-qualification learning in many places. While both ensure that learning is completed and recorded, CE has a focus on pure participation at education or training events, and recording hours of education received. CPD, however, is a cyclical process allowing the participant to reflect on their needs, plan the learning then take action by completing the learning and then evaluate the impact of these on their practice [7]. Completing CPD therefore incorporates more elements than CE. Some may perceive this as a barrier, as it can require more time involvement [8]. CPD requires more effort from the learner, including documentation, which should be concise to show progress over a time period [9]. In addition, CPD requires application of learning into practice, and evaluation and reflection of this, all of which must be documented to demonstrate the implementation of learning, so CPD portfolios should be designed as tools of support, not burdens to complete [9]. Barriers for completion of CPD include time, resource issues and system constraints [10].

Globally, there is inconsistency in the use of the terms CE and CPD; lifelong learning is another term that could be used. Both CE and CPD contribute to lifelong learning, which is essential throughout the life of any professional, ensuring they are up-to-date with current practices, including skills and knowledge. Therefore, both CE and CPD should focus on health priorities and needs identified at individual, organization or national levels, as a quality assurance measure [11]. Thus, participation in mandatory lifelong learning activities should deliver a quality assurance that knowledge, skills, and behaviors are being maintained to demonstrate competence [8,9]. Therefore, including CPD as part of CE provision will aid the movement towards a learner-led, needs-based model, rather than a time-based model motivated by providers [9]. However, not all countries have a culture or mandatory requirement to complete CE or CPD. 

Various reports have been undertaken to investigate CPD requirements globally. The Pharmacy Society of Ireland (PSI) conducted an international review of CPD models in 2010 [12] and Tran et al. reviewed models of CE/CPD in 2014 [13]. When the International Pharmaceutical Federation (FIP) reviewed CE/CPD in Pharmacy globally in 2014 [7], 66 countries were investigated and only 33 had CE/CPD requirements in place in order to maintain registration, showing CE and CPD are used but not widespread. Of those countries where CE/CPD is present, 76% used a “credit system” with 33.3% using a portfolio system. 

Driesen et al. [14] noted that there is no global model in place for lifelong learning. Nevertheless, various models have been outlined for CE or CPD including assessment [15], learning at work [16], reflection [17], peer review [18], and specialization [19]. Formats used include face-to-face, distance learning which includes sent written material to review, and online learning, including webinars or e-learning activities. Models used for CE/CPD differ globally, and also within countries, but no review of these models has been carried out, so currently providers of lifelong learning have no reference of whether any particular model shows better outcomes, or is more preferred by pharmacists. Where there are no CE/CPD requirements in a country, pharmacists may still want to engage in learning activities, so identifying current approaches used may benefit those introducing models in the future.

Bruno et al. [20] point out that despite the differences seen in different countries, the improvement of patient health is the key goal that binds all practitioners. The Irish review recognized that a CPD model must focus on practitioner development to ensure that skills and knowledge are built upon throughout a career, whilst recognizing different jobs in different career settings, with a primary focus on patient care. It also showed that a balance of activities is needed to achieve CPD and the focus should be on outcomes, rather than inputs [12]. When comparing CE and CPD, it has been noted that CPD offers a greater return of investment compared to CE, as there is a greater focus on context and application [10,11]. It has also been noted that CPD must facilitate changes in behavior to support advancement of pharmacy practice [21]. A study by Driesen et al. [14] noted that CPD has had increasing popularity in countries that have a tradition of lifelong learning, with associated behavioral change. Another study by McConnell et al. [22] echoed this, showing that participants noted greater practice improvement after CPD compared to those participating in CE. In 2018, Wheeler et al. [8] also noted the benefit of CPD on practice over CE. Any education program that a pharmacist participates in should support assurance of competency to practice and increase application of knowledge into practice for the benefit of service users [9].

To our knowledge, there is no published systematic review of peer reviewed research evaluating the various models used or the format of CE and CPD interventions globally. Therefore, considering global partnerships and movement of individuals who will be required to keep up-to-date wherever they work, the primary aim of this study was to identify the differing formats or models used by pharmacists for CE/CPD globally. The secondary aim was to identify preferences of pharmacists, in relation to the variety of formats or models used to fulfil mandatory requirements, in order to support future planning of lifelong learning events.

## 2. Materials and Methods 

### 2.1. Design of Study

The methodology used for completion of the systematic review followed the recommendations made from the Preferred Reporting Items of Systematic Reviews and Meta-Analyses (PRISMA) [23].

### 2.2. Criteria for Considering Studies for this Systematic Review

The review was carried out between April and May 2018 to identify any papers published between 1995 and the end of March 2018. Exclusion criteria included not being available in the English language, not being available as a full-length article, not dealing with human subjects, and studies focusing on undergraduate pharmacists. 

### 2.3. Search Strategy

PubMed, Science Direct, and Web of Science were used for gaining papers. Additional studies were also identified from found paper references. Additional articles were identified using Google Scholar, and the university library search engine. Search terms included pharmacist (Title/Abstract), continuing professional development (Title/Abstract), continuing education (Title/Abstract), lifelong learning (Title/Abstract), education and training (Title/Abstract), model (Title/Abstract), framework (Title/Abstract), content (Title/Abstract). 

### 2.4. Data Extraction

Studies were identified that would be looked at further if their title suggested they focused on the aims of the study. Titles were removed if they fulfilled any of the exclusion criteria. Further to this initial screening, full papers were reviewed and removed where no results were seen, or where the primary objectives of the paper did not investigate models or formats of CPD or CE or where the primary objective of the paper did not investigate pharmacist preferences for learning. Studies looking at beliefs, motivators, and barriers to learning were excluded due to a previous review [10]. No grey literature was included in this particular review, which may have resulted in some literature being missed. 

### 2.5. Quality Assessment

The Best Evidence Medical Education Collaboration (BEME) gives guidance on ranking articles, according to strength and importance, [24] which was utilized. 

A summary of the studies found was made capturing author and year of publication, demographics of the study, method used for data collection, objectives of the study, and key findings. Comments were also then made by the lead researcher to emphasize the importance of the study, prior to ranking according to BEME criteria.

Due to this study being a systematic review of previously published papers, ethical approval was not required.

## 3. Results

**Figure 1:**
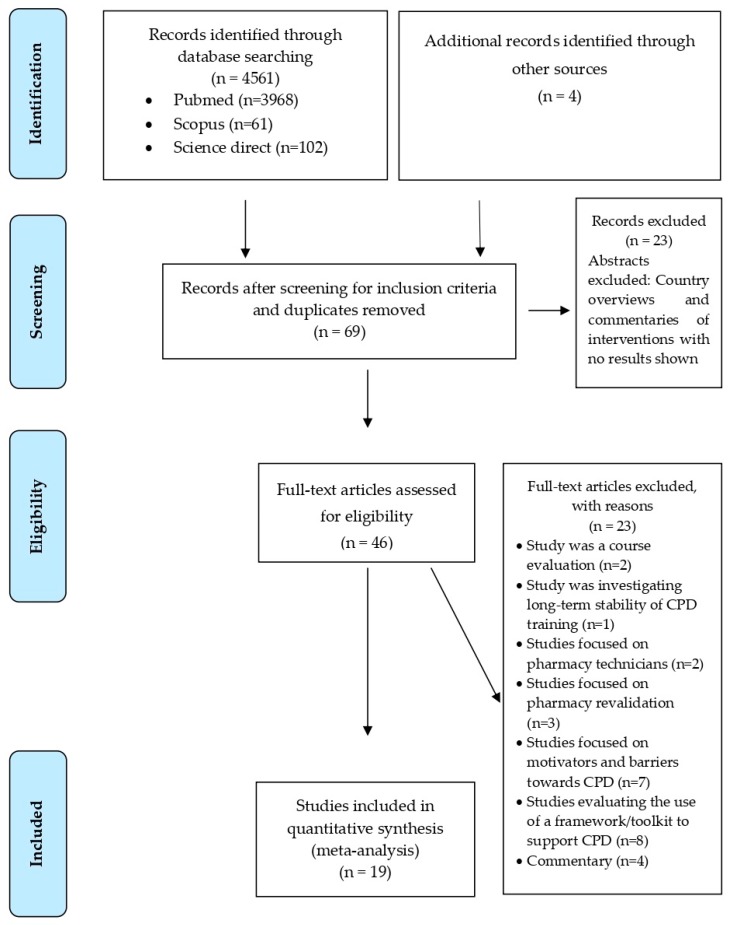
Flowchart of search strategy and article selection.

Using the criteria for the initial search identified 4561 papers. In total, this resulted in 69 studies being identified for further screening, with 19 remaining that were subsequently included in this systematic review. Figure 1 shows the full process of the search, with Table 1 showing the studies identified. Table 1 shows all the included studies, outlining the demographic characteristics of the study, the method of data collection, the objectives of the study, key findings, comments on the study, and suggested BEME scores.

### 3.1. Demographic Characteristics

All articles were published between 2005 and 2014. One included study was a systematic review, so is not included in the demographic data review here [39]. The remaining 18 studies came from seven different countries, with six coming from the United States of America [9,22,35,36,37,40], three from Canada [25,26,33], two each from the United Kingdom [27,38], Belgium [28,29], and Australia [31,41], and one each from Egypt [34], United Arab Emirates [30], and Qatar [32]. The participant number varied by study, from nine to 4140. Of the studies, seven had up to 50 participants [25,26,27,28,31,37,38], five had between 50 and 100 [9,22,35,40,41], and six had over 100 participants [29,30,32,33,34,36]. All of the studies with over 100 participants utilized a survey as their data collection method. An additional four studies also used a survey [9,22,35,40]. Focus group was used as a solo tool in three studies [26,28,31], with course evaluation used three times [25,37,41]. One study used multiple forms of data collection; feedback, ranking, focus group and interview [38]. Interview as a solo method was used once [27]. Pharmacists were the main target of all studies, with three studies targeting community pharmacists [28,29,31], and two targeting hospital pharmacists [27,41]. 

### 3.2. Formats and Models of the Study

Of the 19 included studies, four of the studies evaluated of face-to-face CPD interventions [9,22,26,41], with an additional four evaluating online learning, focusing on CE [33,35,39,40]. One study evaluated a blended learning approach [37], with three involving a review of tools to support CPD completion [27,36,38], and the remainder being surveys to establish preferences for lifelong learning. 

Three articles focused on the shift from CE to CPD [22,25,32]. The remainder of the articles looked at mixture of CE and CPD; CE was the focus of 10 studies [28,29,30,31,33,34,35,37,40,41], with CPD being the focus in 4 [9,26,36,38]. Interestingly, articles from the USA showed a mixture of CE and CPD, due to regional differences in legislation. Other countries focused on the current process used in the country where the intervention took place.

### 3.3. Preferences of Pharmacists

Newer studies focused on the use of technology in educational interventions. The four face-to-face interventions reviewed [9,22,26,41] took place between 2006 and 2014, whereas all online learning interventions took place between 2012 and 2014 [33,35,39,40], showing later introduction of online provision, and future opportunities. However, it is seen that technology can still be an issue in some places [34,39] and face-to-face learning is still preferred where this option is given [28,30,37]. It is noted that all online events were CE events. Satisfaction in participation in online learning did not differ whether participation was in single or multiple sessions [35]. However, it is seen that participation in webinars is more satisfactory when completed live, rather than after the event, in terms of application into practice and audio-visual satisfaction [37]. However, completing at a later time allows working at the individuals’ pace, therefore not missing a learning opportunity [34]. Combining face-to-face with technology, such as clickers, to increase engagement showed positive results [41]. Interactive learning was seen as a positive experience [30,31,32,39,40,41]. When using online learning, access to the internet also needs to be considered to ensure participation [32,34]. It is seen that no one size fits all, so multiple forms of CE/CPD may need to be utilized according to the audience [35,37], although this may differ according to demographic groups [29,34]. For example, women had a preference for lectures over workshops and those not interested in lectures were more likely to be over 44, male, and own a pharmacy [29]. Younger pharmacists were more likely to access online programs [34]. Men are concerned about cost and women are concerned about location [34]. Participants who do not have a specific requirement for format appear more motivated to attend learning events [29]. 

### 3.4. Preferences for Structure of Event to Support Future Planning

Prior to preparing a training session, the need to complete a needs assessment was highlighted [26]. Timing of events should also be considered, to ensure participation and limit scheduling conflicts [32,35]. 

When looking at the structure of the event, support is needed in the process of CPD, with clear structure of the event in order to support learning needs [25,36,38]. Professional outcomes or formal requirements for engagement is seen to be important as a driver for attendance [26,28]. Therefore, understanding of these formal processes need to be understood along with the tools to support the completion of CE/CPD records [9,33,38]. Relevance to practice of topics is also highlighted [28,30,34] ensuring opportunities are available for application of learning [27,36]. This is supported by workplace peer mentors [25,31], taking into account individual advantages/disadvantages of work-based learning approaches [27]. A good speaker or facilitator is highlighted as supporting the outcomes of an event [28]. Reflection of personal practice is seen in CPD rather than CE [9,22,25,31].

When looking at outcomes achieved after events, reading articles resulted in practice improvement [33], as did both live and audio-visual events that are used for CE [37]. Increased practice outcomes and patient care were seen after CPD, rather than CE interventions [22,25]. When using a CPD process to plan learning activities, more changes in practice were seen [9]. However, this change in practice was not always measurable [36]. Whilst e-learning increased knowledge and skills initially, there was no evidence to show an increase in long-term knowledge [39].

## 4. Discussion

Although CE and CPD has been mentioned for many years, only limited studies have identified the preferences of pharmacists for participating in lifelong learning, including CE and CPD activities, and what model is the most effective for learning of pharmacists. While literature does exist in other disciplines, pharmacists may have specific needs that need to be addressed. This review highlights elements of preference, but no clear model of preference. It should also be noted that all papers reviewed came from countries with mandatory systems of CE/CPD in place [7,13,14].

Attewell et al. [42] showed that some pharmacists did not understand the relevance of CE/CPD once their careers were progressing, so many were not fully engaged. As seen in the review, the quality and facilitation of delivery impacts on participation [10,43] along with understanding of CE/CPD and technical problems [10] although external factors do impact on CE/CPD accomplishment [44]. The studies showed that participation increased where there was a mandatory requirement to take part, echoing that tools to capture learning need to be easy to complete, to ensure that assurance of competence can be demonstrated. Echoing other available studies [8,9], participants in studies identified, found the process of completing CPD documentation to be a barrier. Timing of events was highlighted as a barrier to attendance in this study [32,35]. These should be planned in accordance with local needs, as seen in a previous study, to increase participation [45].

The review outlined that clear outcomes for the learning and how it can be applied into practice and benefit the workplace are essential to facilitate interest in the learning [46,47]. Having confidence in the format and process of learning will increase participation, as well as having support in the workplace. Power et al. (2011) [48] noted that hospital pharmacists are more confident in the process than community pharmacists. It is also important to recognize that a range of learning formats should be used and topics need to be targeted to those individuals motivated to learn [49]. Studies showed [28,29,30,34] that topics should be relevant to practice, predominately clinical, and focus on therapeutic areas. Topic has previously been seen as a key driver for attendance [45]. When correlating topic choice and format preference, Driesen (2008) [29] identified that face-to-face is preferred for topics where participants have the least knowledge.

The review showed that face-to-face activity is preferred, where possible. Face-to-face activity allows student and instructor interaction and immediate feedback, although this is more time and resource intensive. Previous research by Schindel et al. (2012) shows that pharmacists value face-to-face training [49]. With a variety of face-to-face methods available, it is vital to give participants choice, ensuring information is presented in a way that is tailored to their learning style [48].

Distance learning, in addition to online learning, can provide a more flexible approach for pharmacist development, as it does not yet replace fully traditional face-to-face learning [40]. Distance learning was seen as a format starting in 2007 [28,29]. The articles relating to online learning were published over a two-year period, whereas the articles relating to face-to-face were published over an eight-year period. However, none of the studies identifying practice outcomes found an increase in learning outcomes as a result of online provision, and a study from 2013 [37] comparing face-to-face versus online showed a preference for a face-to-face approach. Although initially a cost may be incurred from creating the learning, cost savings can be seen from using online learning [50]. Combining formats has the potential to also increase uptake in activity due to flexibility [43]. When using a blended approach, combining distance and face-to-face learning, gender was not associated with outcomes, although those with a preference for online learning showed higher scores for perceived learning, learning application, and motivation [51]. However, a study by Lim et al. (2007) looking at perceived and actual learning, no difference was found between online and blended learning approaches [52]. 

As further identified in the review, due to the variety of formats and no consistent model, it is hard to clarify results or to identify a perfect activity, as also identified in previous research [53,54]. This shows that lifelong learning interventions do need to be individualized, and these may change with new models being introduced. As outlined in the introduction, various models have also been identified since the end date of this review [18,19]. Future work should focus more on online learning and new and emerging mediums, such as social media. A systematic review of media methods of delivery did not identify any current media method as the most effective [55].

Pharmacists learn differently, and this is influenced by multiple factors. Although this study has identified elements to support the perception of a good program, e.g., topic and facilitator, quality assurance of programs overall was outside of the scope of this review. Interestingly, none of the studies mentioned quality assurance of programs delivered. However, ensuring programs are fit for purpose to ensure patient and health outcomes is important. 

As seen in the study, CPD has increased practice outcomes over CE, with increased reflection and application of learning into practice. CPD, therefore, when compared to CE, supports the quality assurance of competence as evidence of application of knowledge, and reflection of this must be demonstrated. This demonstration of application links to higher levels of learning theories, such as Kirkpatrick or Blooms taxonomy. Supporting providers to create programs that help the participant to learn, reflect, apply, and then evaluate practice is encouraged. Using Kirkpatrick or Blooms taxonomy would aid the creation of uniform learning measures, to support evaluation of practice outcomes [55,56]. In future work, learning evaluation models could be explored in more detail to support the creation of educational programs, targeting all stages of learning, supporting the analysis of information, evaluation of evidence and planning, and carrying out activities that will lead to behavioral change. Effective lifelong learning activity would incorporate all six elements listed in Blooms taxonomy [57]. Future studies should also investigate quality assurance aspects of programs, including all stages of learning, from reflection through to application of knowledge and not just focus on preferences.

Limitations for this review are that there are some missing articles, due to no grey literature being reviewed. A cascading process would be used in future reviews. Due to the limited number of papers found, it is hard to draw any conclusive answers relating to difference in preference for CPD versus CE, although it is noted that all online studies found were related to CE provision.

## 5. Conclusions

Although an increasing amount of technology is being utilized, face-to-face learning is still preferred. Interactive learning should be used where possible, and multiple formats, to reflect preferences of different learners. There is a need for a structured approach to the planning and learning event itself, for learners to continue to benefit from support to achieve the CE or CPD process, and regulatory requirements. The transition globally towards CPD, in comparison to CE, is a positive move encouraging reflective practice, and application of learning, with increased outcomes being seen from CPD interventions. Organizers of CE and CPD interventions must identify their audience preferences in order to select the appropriate model for their chosen intervention.

## Figures and Tables

**Table 1:** Summary of studies showing formats used to support continuing professional development (CPD) or continuing education (CE) or pharmacist preferences.

Study Author and Year Research Completed	Study Demographic	Method of Data Collection	Objectives of Study	Key Findings	Comments on Importance of the Study	Best Evidence Medical Education Collaboration (BEME) Score
Austin et al., 2005 [25]	42 pharmacists; Ontario, Canada	focus group (2003)	Examine pharmacists’ attitudes, behaviors and preferences towards CPD	4 themes identified supporting definitions and evolution of CE to CPD supported by workplace learning and peers	The study supports the shift from CE to CPD, supported by peer mentors	Strength 2 Importance 2a and 3
Austin et al., 2006 [26]	47 pharmacists who had completed peer assessment but had not met satisfactory standards; Ontario, Canada	self-assessment and course evaluation (2002)	To develop a professional skills enhancement workshop, to support and maintain competence	Over 90% were positive that the workshop supported current standards of pharmacy practice. When developing CPD programs needs of the practitioners need to be identified	The study confirms the need for needs-assessment prior to creation and running of a course, along with clear expectation of what is needed to meet professional standards	Strength 3 Importance 2a and 3
Swallow et al., 2006 [27]	9 hospital pharmacists; Durham and Tees, UK	interview (2003)	To analyze knowledge gain through the use of a portfolio and the use of this knowledge in informing clinical decision making and practical services	“Socialized learning” and “learning amplification”, were key themes and the findings emphasized the importance of recognizing the advantages/disadvantages of work based (socialized) learning approaches	The study identifies that external factors can affect knowledge utilization	Strength 1 Importance 1
Driesen et al., 2007 [28]	39 community pharmacists; Belgium	focus group (2004)	To examine how current CE courses can be optimized, determine interest in distance learning, and identify what pharmacists think about mandatory CE	Live courses are supported by good speakers, extensive course notes, and focus on topics relevant to practice. Interest in using distance learning was limited. For non-attenders, a formal requirement of engagement is needed, although live courses are preferred	The study identifies that face-to-face learning is preferred, and motivation and incentives are needed for some to engage	Strength 2 Importance 2a and 4a
Driesen 2008 [29]	1032 community pharmacists; Belgium	survey (2003)	To profile pharmacists based on their preferences for CE formats, and association with motivation to attend courses, preferences for topics and demographic traits.	Older men had the greatest interest in distance learning, did not prefer lectures, and were motivated by material incentives. Those pharmacists who preferred lectures as well as workshops showed the highest intrinsic motivation to engage in CE. Pharmacists preferring lectures but not workshops were more likely to be women and showed a dislike for active involvement in CE.	The study identified that different demographics may have different preferences, but there is not a one-size-fits-all model	Strength 4 Importance 3
Hasan 2009 [30]	132 pharmacists; UAE	survey (2009)	To determine the type and format of CE pharmacists prefer to attend and effectiveness	Interactive workshops were recognized as the most favorable format for CE with computer and internet-based formats also ranking highly, followed by live-in person and printed material-based programs. Pharmacy practice and disease management were preferred topics.	The study showed that face-to-face is preferred with topics relevant to practice being preferred	Strength 2 Importance 2a
Mc Namara et al., 2009 [31]	15 community pharmacists; Australia	teleconference focus group (date not given for intervention)	To identify learning preferences for CE and identify issues with the integration of these preferences into contemporary models of CE delivery	Interactive and multidisciplinary CE were preferred, linking to adult learning principles using problem-based learning. Engaging in CPD was valuable to promote reflective learning.	The study identified that principles of adult learning need to be taken into account, along with the ability to work with peers	Strength 3 Importance 3
Wilbur 2010 [32]	134 pharmacists: Qatar	online survey (2008)	To determine CE needs, preferences and attitudes prior to implementation of the first country-wide CPD program	In the past 2 years, 25% had not attended any live local educational programs with barriers including poor timing and excessive workload. Most pharmacists preferred interactive CE program formats. A third preferred delivery in Arabic. A large number had limited or no internet access at work. The majority were motivated to achieve CPD	The study identified that there is positive motivation towards CPD, but consideration needs to be given towards delivery, regarding language and technology	Strength 2 Importance 1
Dopp et al., 2010 [9]	57 pharmacists; 5 states in the USA	pre and post study survey (date not given for intervention)	To determine whether using a structured tool would support CPD completion compared to control subjects.	Significant outcomes from the CPD stages of reflect, plan, act, evaluate, and record were found between matched study subjects and study and control group comparisons	The study identified that training and support is needed to support the utilization of a CPD tool	Strength 4 Importance 4a
Hasan 2009 [30]	132 pharmacists; UAE	survey (2009)	To determine the type and format of CE pharmacists prefer to attend and effectiveness	Interactive workshops were recognized as the most favorable format for CE with computer and internet-based formats also ranking highly, followed by live-in person and printed material-based programs. Pharmacy practice and disease management were preferred topics.	The study showed that face-to-face is preferred with topics relevant to practice being preferred	Strength 2 Importance 2a
Mc Namara et al., 2009 [31]	15 community pharmacists; Australia	teleconference focus group (date not given for intervention)	To identify learning preferences for CE and identify issues with the integration of these preferences into contemporary models of CE delivery	Interactive and multidisciplinary CE were preferred, linking to adult learning principles using problem-based learning. Engaging in CPD was valuable to promote reflective learning.	The study identified that principles of adult learning need to be taken into account, along with the ability to work with peers	Strength 3 Importance 3
Wilbur 2010 [32]	134 pharmacists: Qatar	online survey (2008)	To determine CE needs, preferences and attitudes prior to implementation of the first country-wide CPD program	In the past 2 years, 25% had not attended any live local educational programs with barriers including poor timing and excessive workload. Most pharmacists preferred interactive CE program formats. A third preferred delivery in Arabic. A large number had limited or no internet access at work. The majority were motivated to achieve CPD	The study identified that there is positive motivation towards CPD, but consideration needs to be given towards delivery, regarding language and technology	Strength 2 Importance 1
Dopp et al., 2010 [9]	57 pharmacists; 5 states in the USA	pre and post study survey (date not given for intervention)	To determine whether using a structured tool would support CPD completion compared to control subjects.	Significant outcomes from the CPD stages of reflect, plan, act, evaluate, and record were found between matched study subjects and study and control group comparisons	The study identified that training and support is needed to support the utilization of a CPD tool	Strength 4 Importance 4a
McConnell et al., 2010 [22]	91 pharmacists; Denver, USA	Online survey at enrolment and after 10-months of follow up study	To assess effects of CPD compared to CE on perceptions of factors relating to practice	Participants of CPD, rather than CE, post-intervention, identified better interactions with other healthcare colleagues and had initiated work changes. In addition, they identified patient care had improved along with professional knowledge and skills. However, time was more of a barrier	The study showed that CPD had positive outcomes on practice compared to CE	Strength 5 Importance 2a and 3
Budzinski et al., 2012 [33]	4140 completed surveys from 67 emails to hospital pharmacist, community pharmacist or pharmacy student; Canada	Questionnaire developed from Information assessment method sent via email (August 2008 to May 2009)	To assess the use of an electronic knowledge resource to record CE activities and identify educational needs	Pharmacists who had read the electronic knowledge resource attributed what they had learnt to practice improvement, learning and motivation to learn more	The study confirms that the use of e-portfolios or questionnaires to record learning is an effective method that can be used to support CE, as they are easily trackable and easy to complete	Strength 4 Importance 4b
Mohamed Ibrahim 2012 [34]	359 pharmacists; Cairo, Egypt	Questionnaire (2010)	To determine CE preferences of pharmacists prior to implementation of a compulsory CE system	Therapeutics and clinical skills were preferred topics. Community pharmacists had attended less CE events than their hospital colleagues. However, hospital pharmacists reported less satisfaction than community pharmacists with CE. Common barriers were cited in addition to some related to technology and employers.	The study identifies the need to be flexible and that there is no one size-fits all approach	Strength 4 Importance 1
Buxton 2012 [35]	50 practicing pharmacists; Wisconsin, USA	survey (2011)	To identify satisfaction with CE webinars and evaluate reasons for enrolment	Whether 1 or more webinars had been completed satisfaction was positive, and no differences were found in motives for enrolment between those only completing 1 or multiple webinars	The study identified that limited number of completions was a concern and that there was a need to address scheduling conflicts and identify other deterrents to participation	Strength 2 Importance 1
Trewet and Fjortoft 2013 [36]	105 pharmacists; USA	3 surveys (2010)	To evaluate the effectiveness of tools designed to support the pharmacist through a CPD process at a national meeting.	Nearly all the test groups reported successful application of learning and achieving their designed learning plan (87%) however practice changes were implemented in more than half of the test groups after using a CPD process to plan their learning activities. There were no significant differences among groups regarding the outcome measures	The study identifies that using a structured CPD approach is useful to support learning outcomes, and incorporating CPD into education events can support practice change	Strength 3 Importance 3
Buxton and DeMuth 2013 [37]	29 pharmacists; Wisconsin, USA	Course evaluation survey (date not given for intervention)	To examine perspectives of a CE program delivered live or via a simultaneous webcast	Whilst both groups were satisfied with the presentation from an audio-visual perspective and the ability to put the learning into practice the live group were significantly more satisfied with the overall learning experience	The study identifies that although a positive experience and a useful alterative to physical attendance, webcasts do not fully replace the experience of being live at a learning event	Strength 4 Importance 1
Donyai et al., 2013 [38]	35 pharmacists; United Kingdom	feedback (n=5), ranking (n=7), focus group (n=6), interview (n=17)	To develop and validate a framework to select CPD activities that are relevant to their work and produce a score sheet to make it possible to quantify CPD impact and relevance	The framework’s content validity index was 0.91. Feedback about the framework related to 3 themes of penetrability of the framework, usefulness to completion of CPD, and advancement of CPD records for revalidation	The study identified the importance of following a structure to support CPD completion	Strength 4 Importance 2b
Salter et al., 2014 [39]	17 studies including pharmacist or pharmacy student	Systematic review (2010)	To examine the quality of e-learning effectiveness and identify success measures	While e-learning effectively increases knowledge and is a highly acceptable format, there is limited evidence that e-learning effectively improves skills or professional practice and no evidence that it can be used to increase long term knowledge	The study identifies that, although a useful tool, e-learning has limited use in long term acquisition of knowledge	Strength 5 Importance 1
Buxton et al., 2014 [40]	82 pharmacists; Wisconsin, USA	50 question online survey (2012)	To evaluate pharmacists’ satisfaction of a CE program offered as either synchronous or asynchronous webinars	Whilst both groups were satisfied with the content of the program the asynchronous group were more satisfied with multiple aspects of the learning program	The study identifies that when not physical able to attend an event, participants would rather access this in their own time and at their own pace	Strength 3 Importance 1
Grzeskowiak et al., 2014 [41]	60 hospital pharmacists; Australia	utilizing and evaluating clicker use throughout presentation (2012)	To evaluate the use of clickers as a potential for an engagement tool in CE activities during a face-to-face event	Attendees were positive about the use of clickers and their positive use in engagement, and advocated their future use	The study showed that using different technologies can increase engagement in learning activities	Strength 4 Importance 1
